# Changes in geometric configuration and biomechanical parameters of a rapidly growing abdominal aortic aneurysm may provide insight in aneurysms natural history and rupture risk

**DOI:** 10.1186/1742-4682-10-67

**Published:** 2013-12-05

**Authors:** Nikolaos Kontopodis, Eleni Metaxa, Yannis Papaharilaou, Efstratios Georgakarakos, Dimitrios Tsetis, Christos V Ioannou

**Affiliations:** 1 Vascular Surgery Department, University Hospital of Heraklion & University of Crete Medical School, PO Box 1352, Heraklion, Crete 711 10, Greece; 2 Institute of Applied and Computational Mathematics, Foundation for Research and Technology-Hellas, Heraklion, Greece; 3 Vascular Surgery Department, “Demokritus” University of Thrace Medical School, Alexandroupolis, Greece; 4 Interventional Radiology Unit, University of Crete Medical School, Heraklion, Crete, Greece

**Keywords:** Peak wall stress, AAA Morphometrics, AAA biomechanics, Rupture risk

## Abstract

**Background:**

Abdominal aortic aneurysms (AAA) are currently being treated based on the maximum diameter criterion which has often been proven insufficient to determine rupture risk in case of every AAA. We analyzed a rare case of an AAA which presented an extremely fast growth focusing on biomechanical determinants that may indicate a high risk profile. The examination of such a case is expected to motivate future research towards patient-specific rupture risk estimations.

**Methods:**

An initially small AAA (maximum diameter: 4.5 cm) was followed-up and presented a growth of 1 cm in only 6-months of surveillance becoming suitable for surgical repair. Changes of morphometric characteristics regarding AAA, thrombus and lumen volumes, cross-sectional areas, thrombus maximum thickness and eccentricity, and maximum centerline curvature were recorded. Moreover biomechanical variables concerning Peak Wall Stress, AAA surface area exposed to high stress and redistribution of stress during follow-up were also assessed.

**Results:**

Total aneurysm volume increased from 85 to 120 ml which regarded thrombus deposition since lumen volume remained stable. Thrombus deposition was eccentric regarding anterior AAA segment while its thickness increased from 0.3 cm to 1.6 cm. Moreover there was an anterior bulging over time as depicted by an increase in maximum centerline curvature from 0.4 cm^-1^ to 0.5 cm^-1^. Peak Wall Stress (PWS) exerted on aneurysm wall did not change significantly over time, slightly decreasing from 22 N/cm^2^ to 21 N/cm^2^. At the same time the area under high wall stress remained practically constant (9.9 cm^2^ at initial vs 9.7 cm^2^ at final examination) but there was a marked redistribution of wall stress against the posterior aneurysmal wall over time.

**Conclusion:**

Aneurysm area under high stress and redistribution of stress against the posterior wall due to changes in geometric configuration and thrombus deposition over time may have implications to aneurysms natural history and rupture risk.

## Background

Abdominal aortic aneurysm (AAA) is a major health problem, becoming more common with ageing of the population and aneurysmal disease is the 13th leading cause of death in Western societies [1]. The most catastrophic complication of this condition is rupture, which in the past occurred in up to a third of patients left untreated with an overall mortality of 80% [2]. To prevent this devastating outcome, elective AAA repair has been performed successfully for several decades and is currently associated with mortality rates < 3% [3]. According to current guidelines, large aneurysm size as represented by a maximum diameter ≥ 5.5 cm and rapid growth rate ≥ 1 cm/year foretells a high risk of rupture and therefore sets the indication for surgical intervention. On the other hand small aneurysms should be followed with ultrasound or computed tomography (CT) to detect expansion [3,4]. Nevertheless it is well established that such patients may also experience the catastrophic results of rupture with some studies postulating that as high as 10% of all ruptured AAAs have a maximum diameter less than 5 cm [5].

Since aneurysm size and growth rate have often been proven inaccurate to predict each AAA’s evolution there is a considerable effort for other markers to be found to assist in rupture risk estimation. According to the biomechanical approach rupture represents a material failure of the degenerated AAA wall to withstand the stress exerted on it due to systemic pressurization [6]. Wall stress depends on each AAA unique geometric configuration and therefore it might provide a more accurate rupture risk estimation compared to the universal maximum diameter criterion [7]. It has been proposed that Peak Wall Stress (PWS) exerted on the aneurysmal wall not only is significantly different between diameter-matched ruptured and non-ruptured AAAs but also differentiates aneurysms with a higher rupture risk over time better than the maximum diameter. Furthermore, the site of rupture has been suggested to correlate with the location of PWS in ruptured AAAs [8-10].

Small AAAs are reported to grow with a mean annual rate of 0.2-0.3 cm [3,11]. We came across a case that presented a far faster enlargement of 1 cm within a 6 month interval that equals an annual growth rate of 2 cm/year making it amenable to surgical repair. The analysis of the geometric and biomechanical profile of such a case is expected to provide insight into AAAs mechanisms of growth and rupture and identify possible predictors of a high risk potential to assist in rupture risk estimations.

## Materials and methods

### Patient demographic and clinical information

A 75 years old Caucasian male patient was diagnosed with AAA during an abdominal ultrasound that was performed for other medical reasons. From the lifestyle risk factors and medical history, the patient was a smoker and he was under medication for chronic obstructive pulmonary disease, diabetes mellitus and arterial hypertension. He had no family history of aneurysmal disease. The incidentally discovered AAA was evaluated with abdominal CT scan and was measured to have an initial maximum diameter of 4.5 cm. After an interval of 6 months a second CT scan was performed to detect aneurysm expansion. A maximum diameter of 5.5 cm was recorded. In order to ensure accurate expansion rate determination, AAA maximum diameter and growth rate were determined using orthogonal measurements at both CTs, avoiding possible uncertainties that could have arisen if axial measurements were obtained [12]. According to current guidelines the patient underwent open surgical repair of the AAA to eliminate risk of rupture. The postoperative course was uncomplicated and the patient was discharged from hospital six days after surgery presenting no complications during follow-up.

Ethical approval for this research was obtained from the institutional review board (reference number 12275/15.01.2013). Written informed consent was obtained from the patient for the publication of this report and any accompanying images.

### Data acquisition and geometry configuration

**Figure 1:**
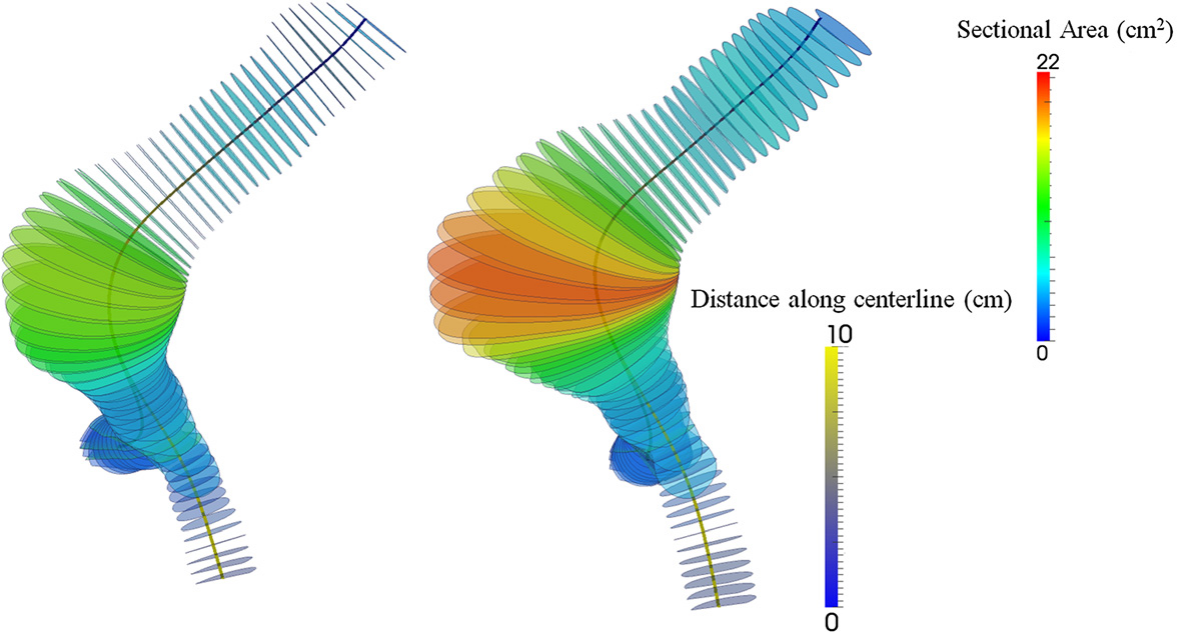
**Cross-sections perpendicular to the centerline with regard to cross-sectional areas for 1st AAA-model (initial examination) and 2nd AAA-model (follow-up).** Maximum sectional areas take values of 22.5 cm^2^ and 15 cm^2^ respectively. Color scale on the centerlines depicts distance along the centerline with 8 cm representing aortic bifurcation.

**Figure 2:**
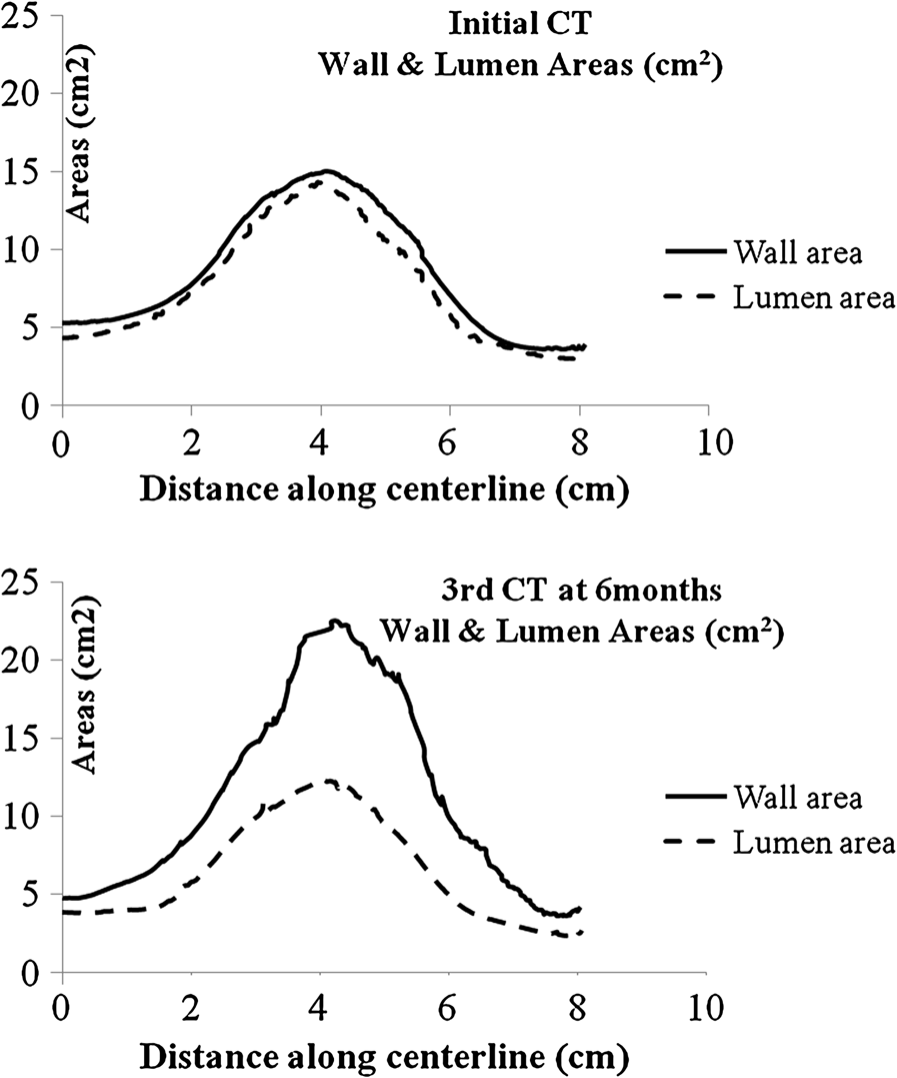
Surface areas of sections vertical to the centerline for total aneurysm wall and lumen for 1st (initial examination) and 2nd AAA-model (follow-up) in relation to distance along the centerline (aortic bifurcation at 8 cm).

**Figure 3:**
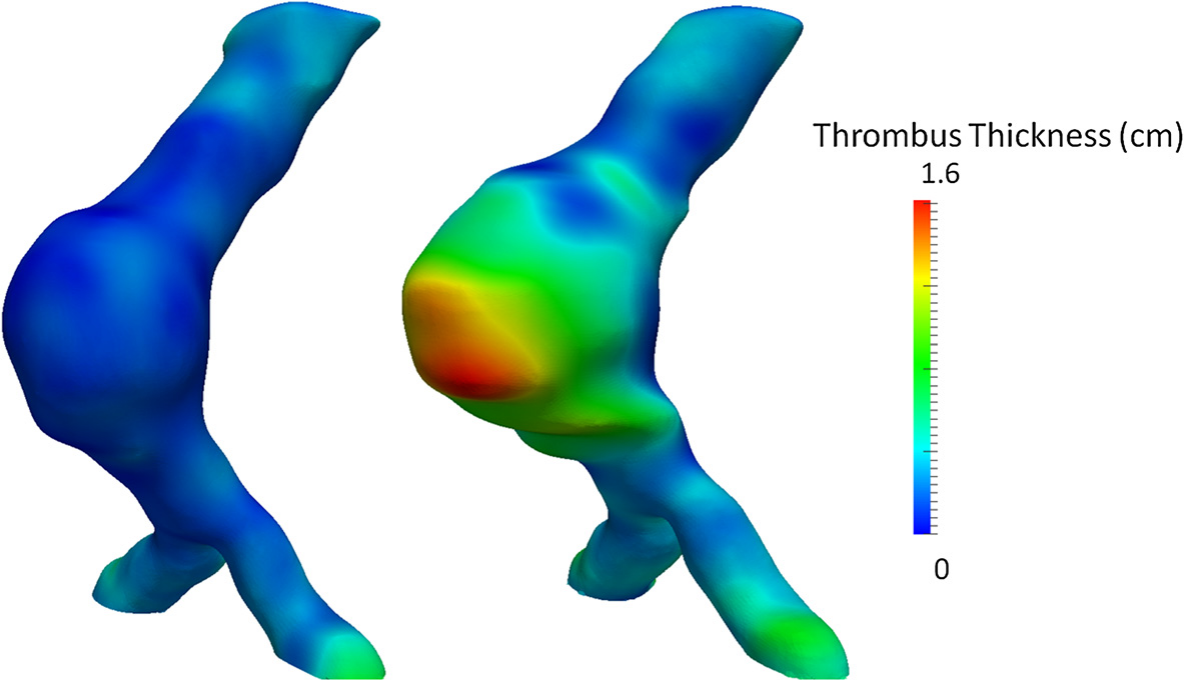
**Thrombus thickness as it is represented for 1st (initial examination) and 2nd AAA-model (follow-up).** The reference values and color scale have been taken with respect to the 2nd model. The corresponding maximum values of ILT thickness are 0.3 cm and 1.6 cm. It is observed that ILT locates anteriorly.

**Figure 4:**
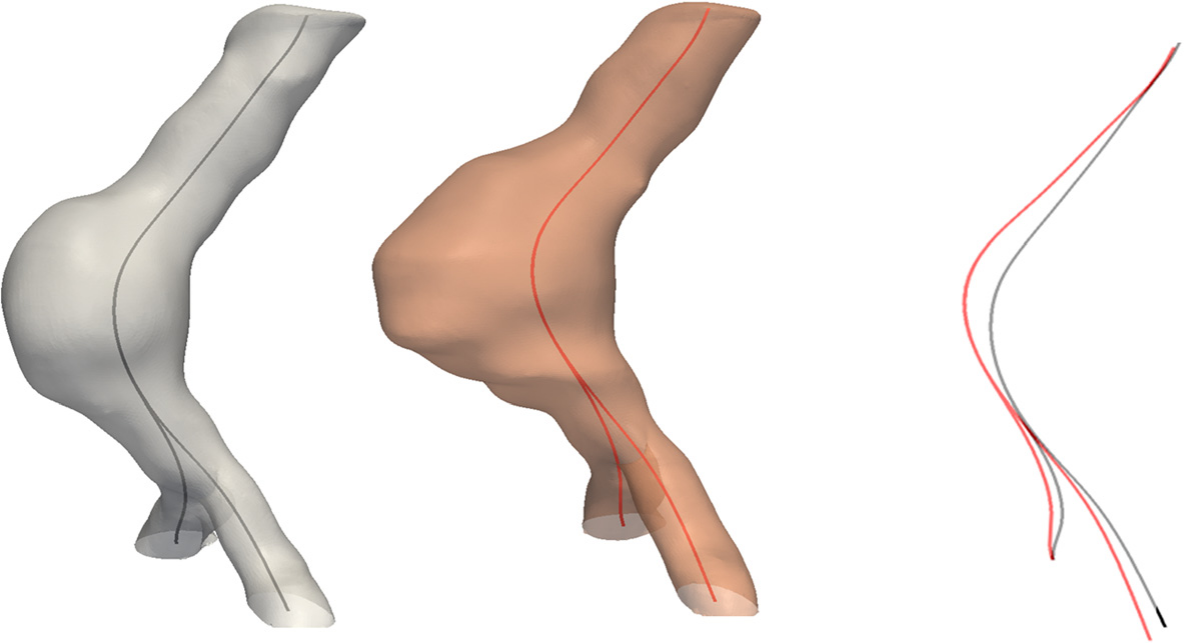
**Increase of maximum centerline curvature during follow-up suggests an anterior bulging of AAA over time.** Centerline at **A**. 1st CT, **B**. 2nd CT, and **C**. Overlapping of the two centerlines.

Contrast-enhanced high-resolution spiral CT angiography was used for AAA evaluation. From 2D images, 3D AAA models were reconstructed using both manual and automatic segmentation for initial and follow up CT scan [13]. For the segmentation process the open source software ITK-SNAP [14] was used. For each model the total aneurysm volume, the lumen and intraluminal thrombus (ILT) volumes were recorded from the most caudal renal artery to the last slice before the aortic bifurcation according to reporting standards on measuring changes in aneurysms size [15]. The open source software *GNU Triangulated Surface* Library 0.7.6 was used for volume measurements. Geometric configuration of the AAA models was assessed using the open source software Vascular Modeling Tool Kit (VMTK) [16]. Centerlines of the aneurysmal wall as well as lumen surfaces were created and used to extract perpendicular cross sections every 1 mm as it can be seen in Figure 1. To determine the aneurysm’s pattern of expansion, cross-sectional area change from initial to follow-up examination was plotted against the distance from aortic bifurcation which is being presented in Figure 2. ILT thickness was also recorded along the aneurysm for both AAA models (Figure 3). Eccentricity of ILT deposition was assessed at the cross section of maximum size, by introducing the Eccentricity Index (EI), which is defined as 1-(Minor Distance/Major Distance) where the Minor and Major Distance refers to the minimum and maximum distance, respectively, between the lumen centerline and the aneurysmal wall boundary. Apparently the zero value would represent a concentric ILT distribution whereas the unity would represent a profoundly eccentric ILT deposition. Finally maximum curvature of the centerline was recorded to evaluate bulging of the AAA wall at both initial and final examination and this is being shown in Figure 4.

### Biomechanical parameters

For the wall stress analysis, a 3D mesh was generated using ICEM CFD (ANSYS Inc., Berkeley, CA, USA). The wall stress analysis was performed with ANSYS 12.0 Workbench (*ANSYS* Inc., Berkeley, CA, USA) and its static structural analysis. For the wall and ILT, neo-Hookean hyperelastic material models were adopted, with the following strain-energy functions respectively:

$$W_{\mathrm{WALL}}=\alpha \left(I-3\right)+\beta {\left(I-3\right)}^{2},\;W_{\mathrm{ILT}}=c_{1}\left(\mathrm{II}-3\right)+c_{2}{\left(\mathrm{II}-3\right)}^{2}$$

where α = 0.174 MPa and β = 1.881 MPa [17], c_1_ = c_2_ = 0.026 MPa [18], are material parameters, I, II are the first and second invariants of the Left Cauchy-Green tensor C, J^2^ = detC. The AAA model was loaded with the patient’s systolic pressure.

The PWS (based on the von Misses criterion) was recorded.

Moreover the aneurysmal surface area exposed to high wall stress was also calculated. For this purpose, a threshold of wall stress was defined. The mean physiological wall stress was computed using an idealized model of infrarenal aorta loaded with intraluminal pressure of 120 mmHg and a diameter of 20.9 mm, which is the mean aortic diameter based on the patient’s age and gender [19]. A 100% increase of the physiological stress was used as a threshold to define high wall stress.

By using the open source software ParaView 3.14 (A. Henderson, ParaView Guide, A Parallel Visualization Application. Kitware Inc., 2007), this threshold was then applied to identify the wall regions exposed to high wall stress, and by integrating their areas, to obtain the total wall surface area under high wall stress. Finally to evaluate the redistribution of wall stress during follow-up, the anterior and posterior site of each AAA were defined by using the z-y plane of the CT scanner coordinates system, and the area under high wall stress located anteriorly and posteriorly were calculated for the initial and final state.

## Results

### AAA geometric characterization

Total aneurysm volume increased between initial and follow-up examination from 85 ml to 120 ml. This volume change matched the increase in ILT deposition since lumen volume remained constant (72 ml and 71 ml for initial and follow up examination respectively) while ILT increased from 14 ml to 50 ml. To spatially examine the aneurysm pattern of expansion, cross sections perpendicular to the centerline were extracted and their surface area was measured. The cross-sectional area of the aneurysm wall progressively grew over time, while the lumen area did not increase but showed slightly greater values in the first model. At the site of maximum size, AAA cross-sectional area was 15.0 cm^2^ for initial and 22.5 cm^2^ for follow-up examination. Corresponding values of lumen cross-sectional areas were 14.3 cm^2^ and 12.4 cm^2^ while those of ILT were 0.7 cm^2^ and 10.1 cm^2^. Cross-sections of the two AAA models are displayed in Figure 1 whereas a graphical representation of the cross-sectional areas with regard to the distance from bifurcation is presented in Figure 2. Maximum AAA and ILT cross-sectional areas were observed at the same distance from aortic bifurcation that was 4 cm for both AAA models. Apparently, maximum ILT thickness was markedly increased during follow-up from 0.3 cm to 1.6 cm and this regarded the anterior AAA portion as shown in Figure 3. EI was initially 0.2 while a value of 0.6 was found for final state representing the eccentric anterior deposition of ILT during follow-up. Maximum curvature of the AAA centerline also increased from 0.4 cm^-1^ to 0.5 cm^-1^, suggesting an anterior bulging over time as represented in Figure 4.

### AAA biomechanical parameters

**Figure 5:**
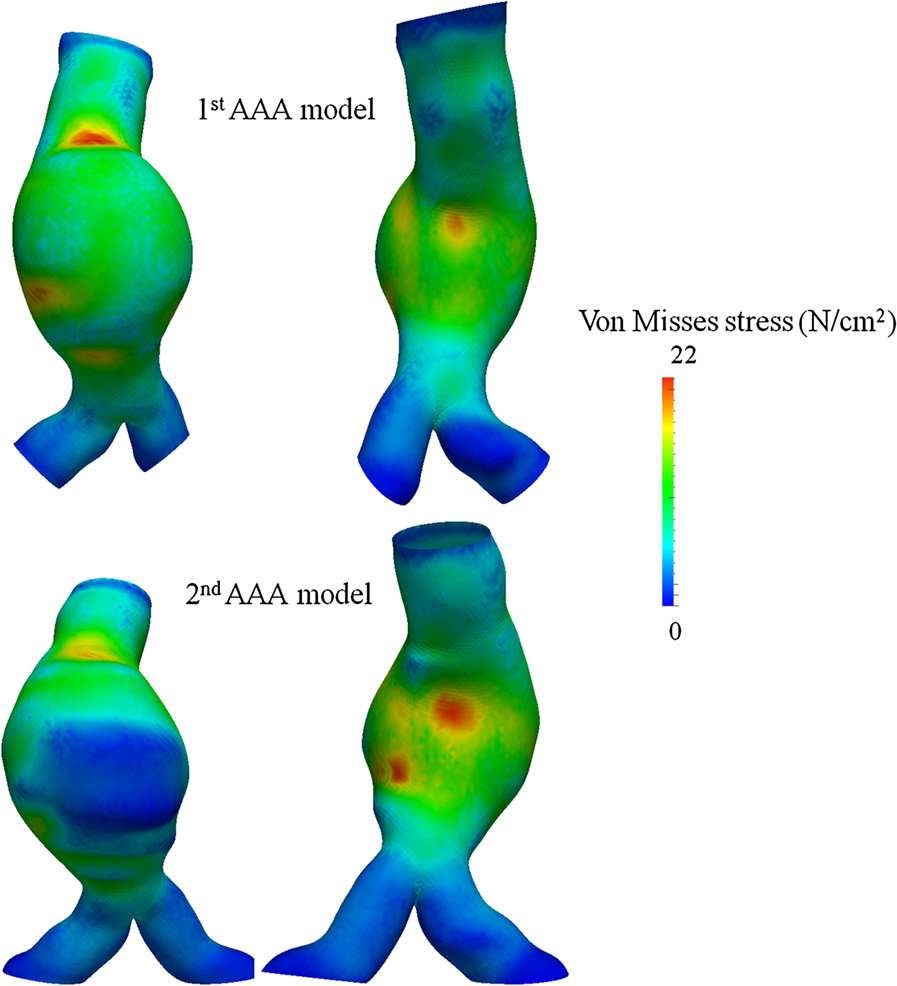
**Anterior and posterior views of wall stress distribution throughout the AAA surface for the 1st (upper row) and 2nd AAA-model (lower row).** A redistribution of wall stress against the posterior aortic wall is noticed.

PWS throughout the aneurysmal surface remained practically constant presenting a slight decrease from 22 N/cm^2^ to 21 N/cm^2^. Wall stress distribution for the two AAA models is presented in Figure 5. Mean wall stress on the idealized aorta was 10 N/cm^2^ and therefore a threshold of 20 N/cm^2^ was used as a cut-off value to define high wall stress. The aneurysmal surface area under high wall stress did not change considerably over time presenting a value of 9.9 cm^2^ during initial and 9.7 cm^2^ during final evaluation. On the other hand a marked redistribution of wall stress throughout the aneurysmal surface over time could be established. Specifically, initially area under high stress was located at the anterior site and the AAA neck, while at final state high stress areas are only present at the posterior AAA wall. Subsequently, total posterior wall area exposed to high stress, was 0 cm^2^ and 9.7 cm^2^ at initial and final states respectively.

## Discussion

We present a rare case of a small AAA that grew unexpectedly fast presenting an increase in maximum diameter of 1 cm in only 6 months of surveillance and becoming eligible for surgical correction. It is well established that rapid AAA expansion indicates a high risk of rupture [3,20]. Similarly to most AAAs that present a preferential anterior expansion of the aneurysmal wall due to limitation of posterior expansion from the vertebral column presence, enlargement was also anterior in our case [21]. By studying this rare case of rapid growth rate, which otherwise presented a rather common geometrical pattern of growth, hypotheses on new potential morphological and biomechanical markers may arise that will give direction to future studies.

An interesting finding is that regarding the AAA under evaluation the PWS did not increase during follow-up, in contrast to previous studies that suggest an association between PWS and growth rate [22]. Furthermore the AAA surface area exposed to high stress did not considerably change as well. On the other hand the location of high stress showed a marked redistribution over time. At the initial AAA state, area under high wall stress was mainly located at the aneurysmal neck which is a very unlikely site of rupture according to autopsy studies [23,24]. On the contrary at the final AAA state, the area exposed to high wall stress was concentrated at the posterior AAA portion, which is the site of rupture for the majority of ruptured AAAs. We hypothesize that augmentation of posterior aneurysmal surface area exposed to high wall stress may increase the likelihood of rupture. Posterior AAA wall regions have been suggested to be thinner and less stiff than those located anteriorly which may lead to a decreased mechanical endurance. This is supported by the results of several autopsy studies that indicate a posterior rupture site for the majority of ruptured AAAs [23-26]. Subsequently a larger aneurysmal surface area located posteriorly needs to be reinforced through arterial wall remodeling to withstand the high stresses and it is therefore more likely for a weak wall spot to be exposed to high stress. Additionally, as pointed out by Mower et al., vasa vasorum and other small blood vessels in regions of increased stress may tend to be compressed and not provide nutrition to the vessel wall [27]. This may cause weakening of the aneurismal wall with impaired repair or remodeling mechanisms which would have an influence on AAAs evolution.

However, the wall stress computation is currently not practical in a clinical setting. It might therefore be more useful to identify relevant morphological markers that would capture the stress redistribution. Such a marker could be the centerline maximum curvature, which has been suggested to greatly influence stresses on the AAA wall usually against the posterior segment [7,28]. Additionally, the thrombus deposition has been proposed to have a biomechanical cushioning effect reducing the stress exerted on the arterial wall [13]. Eccentric anterior deposition of ILT over time could display the non-uniform stress redistribution against the posterior wall and a means to quantify the former could be the EI as introduced in the current analysis. More importantly EI could be easily calculated on a two dimensional plane of the CT data without the need for 3D reconstruction that is not always available in a clinical setting. More studies are needed to establish importance of such observations.

## Conclusion

In this single case of a small AAA with rapid expansion, anterior bulging and eccentric anterior ILT deposition were noticed. These have lead to marked redistribution of wall stress against the posterior wall which has been shown to be the most common site of rupture. It is expected that such information could be useful towards determining AAAs natural history and obtaining an accurate patient-specific AAA rupture risk estimation. Larger scale studies are needed to validate the significance of these findings.

## Competing interests

The authors declare that they have no competing interests.

## Authors’ contributions

NK participated in the conception of research, contributed to data analysis and performed writing. EM also contributed to design, performed measurements and assisted writing the manuscript. YP have contributed to interpretation of data and critically revised manuscript. EG contributed to the analysis and interpretation of data and critically revised manuscript. DT have been involved in drafting the manuscript. CI participated in the design, critically revised manuscript and had overall responsibility. All authors have given final approval of the version to be published.

## Funding

The research project was partially supported by the Action «Supporting Postdoctoral Researchers» of the Operational Program "Education and Lifelong Learning" (Action’s Beneficiary: General Secretariat for Research and Technology), and is co-financed by the European Social Fund (ESF) and the Greek State.
